# Supplementary material for: Excess cases of influenza and the coronavirus epidemic in Catalonia: a time-series analysis of primary-care electronic medical records covering over 6 million people
Source: BMJ Open. 2020 Jul 29;10(7):e039369. doi: 10.1136/bmjopen-2020-039369 (PMC7431772; doi:10.1136/bmjopen-2020-039369)

Excess cases of influenza suggest an earlier start to the coronavirus epidemic in Spain than official figures tell us: an analysis of primary care electronic medical records from over 6 million people from Catalonia

Supplementary material

Supplementary Table 1. ICD-10 codes used to identify influenza and COVID-19 cases

Influenza

J10.1	Influenza due to other identified influenza virus with other respiratory manifestations
J10.89	Influenza due to other identified influenza virus with other manifestations
J11.1	Influenza due to unidentified influenza virus with other respiratory manifestations
J11.2	Influenza due to unidentified influenza virus with gastrointestinal manifestations
J11.89	Influenza due to unidentified influenza virus with other manifestations
J10	Influenza due to other identified influenza virus
J11	Influenza due to unidentified influenza virus

COVID-19

B34.2	Coronavirus infection, COVID-19 (with PCR)
B97.29	Coronavirus as the cause of diseases classified elsewhere
B97.21	SARS-associated coronavirus as the cause of diseases classified elsewhere
J12.81	Pneumonia due to SARS-associated coronavirus.

Supplementary Table 2. ARIMA models fitted

Total	ARIMA(1, 1, 2)	$Y_t = 1.86_{t-1}Y_{t-1} + 1.22e_{t-1} - 0.37e_{t-2}$
Age younger than 15	ARIMA(4, 0, 1)	$Y_t = 432.67 + 1.96Y_{t-1} - 1.17Y_{t-2} + 0.35Y_{t-3} - 1.60Y_{t-4} + + 0.59e_{t-1}$
Age between 15 and 64	RIMA(1, 0, 3)	$Y_t = 759.6 + 0.96 \quad Y_{t-1} + 0.10e_{t-1} - 0.17e_{t-2} + 0.06e_{t-3}$
Age older than 64	ARIMA(1, 1, 2)	$Y_t = 0.49 \quad Y_{t-1} + 0.70 - 0.27e_{t-2}$

Supplementary Figure 1. Observed and expected (with 95% CI) weekly new influenza cases each day after the peak of the 2018-2019 Catalonia influenza season, in the full population and in each age group.

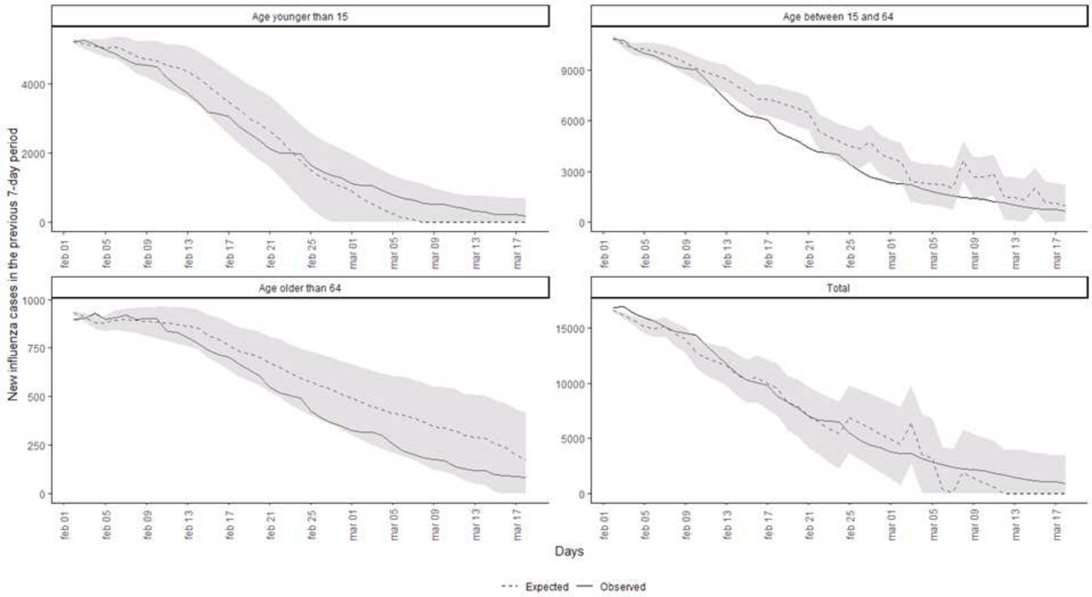

Supplement: Supplementary data [file bmjopen-2020-039369supp001.pdf]
