# Supplementary material for: Excess cases of influenza and the coronavirus epidemic in Catalonia: a time-series analysis of primary-care electronic medical records covering over 6 million people
Source: BMJ Open. 2020 Jul 29;10(7):e039369. doi: 10.1136/bmjopen-2020-039369 (PMC7431772; doi:10.1136/bmjopen-2020-039369)
Supplement: Author's manuscript [file bmjopen-2020-039369.draft_revisions.pdf]

BMJ Open is committed to open peer review. As part of this commitment we make the peer review history of every article we publish publicly available.

When an article is published we post the peer reviewers' comments and the authors' responses online. We also post the versions of the paper that were used during peer review. These are the versions that the peer review comments apply to.

The versions of the paper that follow are the versions that were submitted during the peer review process. They are not the versions of record or the final published versions. They should not be cited or distributed as the published version of this manuscript.

BMJ Open is an open access journal and the full, final, typeset and author-corrected version of record of the manuscript is available on our site with no access controls, subscription charges or pay-per-view fees (http://bmjopen.bmj.com).

If you have any questions on BMJ Open's open peer review process please email info.bmjopen@bmj.com

BMJ Open

Excess cases of influenza suggest an earlier start to the coronavirus epidemic in Spain than official figures tell us: an analysis of primary care electronic medical records from over 6 million people from Catalonia

Journal:	*BMJ Open*
Manuscript ID	bmjopen-2020-039369
Article Type:	Original research
Date Submitted by the Author:	13-Apr-2020
Complete List of Authors:	Coma Redon, Ermengol; ICS, Sistemes d'Informació dels Serveis d'Atenció Primària (SISAP); IDIAP Jordi Gol<br>Mora, Nuria; ICS, Sistemes d'Informació dels Serveis d'Atenció Primària (SISAP); IDIAP Jordi Gol<br>Prats-Uribe, Albert; University of Oxford, Nuffield Department of Orthopaedics Rheumatology and Musculoskeletal Science<br>Fina Avilés, Francesc; ICS, Sistemes d'Informació dels Serveis d'Atenció Primària (SISAP); IDIAP Jordi Gol<br>Prieto-Alhambra, Daniel; University of Oxford, NDORMS; IDIAP Jordi Gol<br>Medina, Manuel; ICS, Sistemes d'Informació dels Serveis d'Atenció Primària (SISAP); IDIAP Jordi Gol
Keywords:	Epidemiology < INFECTIOUS DISEASES, PRIMARY CARE, Public health < INFECTIOUS DISEASES

SCHOLARONE™
Manuscripts

I, the Submitting Author has the right to grant and does grant on behalf of all authors of the Work (as defined in the below author licence), an exclusive licence and/or a non-exclusive licence for contributions from authors who are: i) UK Crown employees; ii) where BMJ has agreed a CC-BY licence shall apply, and/or iii) in accordance with the terms applicable for US Federal Government officers or employees acting as part of their official duties; on a worldwide, perpetual, irrevocable, royalty-free basis to BMJ Publishing Group Ltd ("BMJ") its licensees and where the relevant Journal is co-owned by BMJ to the co-owners of the Journal, to publish the Work in this journal and any other BMJ products and to exploit all rights, as set out in our licence.

The Submitting Author accepts and understands that any supply made under these terms is made by BMJ to the Submitting Author unless you are acting as an employee on behalf of your employer or a postgraduate student of an affiliated institution which is paying any applicable article publishing charge ("APC") for Open Access articles. Where the Submitting Author wishes to make the Work available on an Open Access basis (and intends to pay the relevant APC), the terms of reuse of such Open Access shall be governed by a Creative Commons licence – details of these licences and which Creative Commons licence will apply to this Work are set out in our licence referred to above.

Other than as permitted in any relevant BMJ Author's Self Archiving Policies, I confirm this Work has not been accepted for publication elsewhere, is not being considered for publication elsewhere and does not duplicate material already published. I confirm all authors consent to publication of this Work and authorise the granting of this licence.

For peer review only

"Excess cases of influenza suggest an earlier start to the coronavirus epidemic in Spain than official figures tell us: an analysis of primary care electronic medical records from over 6 million people from Catalonia"

Ermengol Coma [1,2] *, Nuria Mora [1,2] *, Albert Prats-Uribe [3]*, Francesc Fina [1,2], Daniel Prieto-Alhambra[2,3] §, and Manuel Medina-Peralta [1,2]

1- Sistemes d'Informació dels Serveis d'Atenció Primària (SISAP), Institut Català de la Salut(ICS), Barcelona, Spain.

2- Fundació Institut Universitari per a la recerca a l'Atenció Primària de Salut Jordi Gol i Gurina (IDIAPJGol), Barcelona, Spain.

3- Pharmaco- and Device Epidemiology, Centre for Statistics in Medicine - Nuffield Department of Orthopaedics, Rheumatology, and Musculoskeletal Sciences, University of Oxford, UK

*** joint first authors**

§ Corresponding author:

Prof Daniel Prieto-Alhambra
daniel.prietoalhambra@ndorms.ox.ac.uk
Botnar Research Centre, Windmill Road, OX37LD,
Oxford, United Kingdom

For peer review only

Abstract

Objectives: There is uncertainty about when the first cases of COVID-19 appeared in Spain. We aimed to determine whether influenza diagnoses masked early COVID-19 cases and, if so, estimate numbers of undetected COVID-19 cases in a large primary-care database covering >6 million people in Catalonia.

Design: Time-series of influenza and COVID-19 cases, using influenza seasons from autumn-winter 2010-2011 to 2019-2020.

Setting: Primary care, Catalonia, Spain.

Participants: People registered in one of the contributing primary-care practices, covering >85% of the population.

Main outcome measures: Weekly new cases of influenza and COVID-19 clinically diagnosed in primary care.

Analyses:

Daily counts of both cases were computed using the total cases recorded over the previous 7 days to avoid weekly effects on recording practice. Epidemic curves were characterised for seasons with a similar epidemic curve and peak case number as the 2019-2020 season, and used to model predictions for 2019-2020. ARIMA models were fitted, overall and stratified by age, to estimate expected case numbers. Daily excess influenza cases were defined as the number of observed minus expected cases.

Results:

Four influenza season curves (2011-2012, 2012-2013, 2013-2014, and 2016-2017) were used to estimate the number of expected cases of influenza in 2019-2020. Between 4 February 2020 and 20 March 20202, 8,017 (95% CI: 1,841 to 14,718) excess influenza cases were identified.

Conclusions:

COVID-19 cases may have been present in the Catalan population well before the first case was reported on 25 February 2020. COVID-19 carriers may have been misclassified as influenza diagnoses in primary care, boosting community transmission before public health measures were taken. Surveillance of excess influenza cases using widely available primary-care electronic medical records could help detect new outbreaks of COVID-19 in the future.

Key words: COVID-19, Influenza , SARS-CoV-2, surveillance

For peer review only

Article summary

Strengths and limitations of this study

- We used good quality data covering >6 million people and >85% of the Catalan population, obtained directly from primary record records.

- Data had previously been validated against gold-standard influenza sentinel systems.

- We used ecological data and modelled it using data from previous seasons, therefore assuming a direct link between excess influenza cases and the COVID-19 pandemic.

- Excess influenza cases could also have been due to a panic effect, where current coronavirus epidemic, had encouraged people to consult healthcare professionals more frequently and for milder symptoms than usual.

- We lack confirmatory tests or antigenic data for the estimated excess influenza cases, but our results agree with the proportion of influenza samples that tested positive for SARS-CoV-2 in a recent study.

For peer review only

Background

A new infectious disease, now named COVID-19, was identified by Chinese authorities on 7 January 2020 as the cause of an outbreak of pneumonia in Wuhan.[1] Caused by SARS-CoV-2, COVID-19 is asymptomatic in around 1-3% of patients, according to the WHO mission report.[2] Most patients present mild influenza-like symptoms, including fever, dry cough, fatigue, sore throat, dyspnoea, headache, and myalgia.[2,3] Around 20% of symptomatic cases present severe forms of disease that require hospital admission.[1] Older people, men, and those with multiple comorbidities appear more likely to suffer more serious types of COVID-19.[2–6] Conversely, children seem to have a similar probability of infection, but milder and often asymptomatic forms of the disease.[7]

Cases of COVID-19 have grown exponentially and have been reported all over the world. The first three cases in Europe were reported in France on 24 January 2020.[8] The first imported COVID-19 case in Spain was dated 31 January 2020 in La Gomera, and the first in Catalonia reported a month after, on 25 February 2020. The total number of confirmed cases in Catalonia then increased exponentially, with 715 cumulative cases reported by 14 March 2020 and a striking 4,203 on 20 March 2020. Despite these official figures, it is uncertain whether SARS-CoV-2 was circulating in the community before the first official cases. It is difficult to believe, for example, that this airborne infection did not cross the uncontrolled borders between Catalonia and its northern neighbouring country for a whole month. Some have thus speculated that undetected COVID-19 cases may have been categorised as influenza before the first official case was reported in Spain.[9]

Catalonia is fortunate to have a reliable system for influenza surveillance in place. A network of 60 sentinel general practitioners covering 1% of the total population report daily cases of influenza-like illness (ILI) and take samples for differential diagnosis and confirmation of influenza infections in the region.[10] A specialised hospital-based system takes samples from severe hospitalised flu cases.[10] A community-based surveillance system called Diagnosticat also extracts counts of ILI diagnoses from a network of GP health records in real-time, covering 85% of the population.[11] This last approach allows us to examine trends with granularity and to stratify analyses by age and other factors.

As the first cases of SARS-CoV-2 appeared in Catalonia during the influenza epidemic season and the disease shares some symptomatology with influenza, we hypothesised that SARS-CoV-2 could have been circulating in the community before the first confirmed case, resulting in an excess of influenza diagnoses. We aimed to estimate the number of excess influenza cases in Catalonia, globally and by age, and to examine its relationship with the number of clinically diagnosed COVID-19 cases.

For peer review only

Methods

We used a time-series study of influenza and COVID-19 cases. We extracted data from primary-care electronic medical records covering about 85% of the population of Catalonia, around 6 million people. The study period included all influenza seasons from autumn-winter 2010-2011 to autumn-winter 2019-2020.

The key study outcomes were diagnoses of influenza and COVID-19. Daily frequency of influenza cases recorded in primary-care records were obtained from electronic medical records, as is routinely done for the Diagnosticat database.[12]

Diagnosticat is a website that reports in real-time all influenza diagnoses recorded by all general practitioners working at any of the primary-care centres run by the Institut Català de la Salut (ICS). ICS is the main primary-care health service provider in Catalonia and covers about 85% of practices in the region, who all use the same electronic medical record software, ECAP.[13] Diagnosticat includes all clinical influenza diagnosis codes (ICD-10 codes in Supplementary Table 1) and is updated from ECAP daily (since 2010). It presents the frequency of daily influenza cases and the weekly incidence rates per 10^5 population, a unit that allows diagnoses to be compared between territories independently of the number of inhabitants. Influenza data on Diagnosticat has been shown to accurately represent that in a gold-standard source, the sentinel network of influenza infection reports dataset.[11]

The number of COVID-19 clinical diagnoses were extracted and aggregated using the same data source and methods as for influenza diagnoses. Clinical diagnoses of COVID-19 have been recorded in ECAP since 27 February 2020, when bespoke codes were introduced (Supplementary Table 1). Since March 15 2020, Catalan policies have advocated for cases to be defined based on symptoms alone, with serological or PCR confirmation only required when patients are admitted to hospital or are healthcare staff.[14]

Statistical analysis

Daily counts of influenza and COVID-19 cases were computed based on the frequency of cases recorded in the previous 7-day period to avoid weekly effects on recording practice. All influenza seasons in the study period (2010-2011 to 2019-2020) were analysed separately to characterise annual epidemic curves for seasonal influenza.

Influenza seasons with a visually similar epidemic curve and similar peak case number to that of the 2019-2020 season were selected to model predictions for 2019-2020. Auto Regressive Integrated Moving Average (ARIMA) models [15] were fitted to the seasons included in the analysis for the whole population and for three age groups, paedriatic patients (under 15), adults (15-64), and elderly (over 64 years old).

For peer review only

From the fitted time series, the expected speed of decrease in the number of weekly influenza cases for the 2019-2020 influenza season was calculated for each day after the peak. The expected speed of decrease was defined as the difference between the number of influenza diagnoses predicted between the current day t and the previous day t-1, divided by the number of diagnoses predicted for the previous day t-1 ($((cases_t(cases_{t-1})-1)$). Expected influenza cases were calculated using the sequence $G_t = G_0 *\prod^t_{k=1} V_k$, where G_t was the expected influenza cases in the period t, G_0 was the number of cases at the peak and V_k the speed of decrease at day k.

The expected influenza cases for each day on the 2019-2020 season were calculated from the day of the season peak to 20 March 2020, the day of the data extraction. Excess influenza cases were defined as the number of observed minus expected cases, estimated daily as above.

All analyses were performed in R, version 3.5.1. [16]

Patient and public involvement

This research was done without patient involvement. Patients were not invited to comment on the study design and were not consulted to develop patient relevant outcomes or interpret the results. Patients were not invited to contribute to the writing or editing of this document for readability or accuracy.

Results

Previous influenza epidemic curves

Four of the previous nine influenza season curves (2011-2012, 2012-2013, 2013-2014, and 2016-2017) had an epidemic curve and number of influenza cases during the peak similar to the 2019-2020 season, as shown in Figure 1. These four curves were used to estimate the number of expected cases of influenza in 2019-2020. The mean peak number of cases in the included and excluded seasons was 12,762 and 14,680 respectively. The peak number of cases in 2019-2020 was 12,066.

ARIMA models were fitted using the included seasons. Supplementary Table 2 shows the full modelling process and the fitted parameters.

For peer review only

2019-2020 influenza epidemic description

In Catalonia, the 2019-2020 influenza epidemic reached its peak on 4 February 2020, with 12,066 cases in the previous 7 days. Figure 2 shows the evolution of the season compared with past seasons, centred on the day of the peak. By eye, the downwards trend after the peak initially looks very similar to the previous seasons. However, 20 days after the peak, the curve starts to flatten, and the slope slows down. This abnormal pattern in the descending part of the curve differs from the pattern in the previous seasons.

Expected versus observed cases

Figure 3 shows the observed and estimated numbers of weekly new influenza cases (with 95% CI) after the peak of the 2019-2020 influenza season. The estimated expected number of cases were predicted using the selected previous influenza seasons.

In the whole population, observed cases were always greater than expected after the seasonal influenza peak, to some extent. The difference was statistically significant for 23 days between 4 February 2020 and 20 March 2020. Most of these days fell after 8 March 2020, when the difference between observed and expected increased significantly and observed cases remained above the 95% CI band for expected cases for 2 weeks.

There was a greater difference between observed and expected cases among people aged 15-64 years than in both the total population and other age groups, with 25 total days of significant difference. The observed and expected cases diverged earlier than for the total population, separating around 26 February 2020 and remaining significantly different for the rest of the study period.

Observed and expected cases were generally similar in those older than 64 years, until 6 March 2020. Observed cases then quickly rose above expected cases, with the difference becoming significant on 11 March 2020 and remaining so for 9 days, until 19 March 2020.

The shape of the observed cases curve for people younger than 15 years was similar to that for people aged 15-64 years. However, the difference between observed and expected cases was only significantly different for 11 days, between 6 March 2020 and 16 March 2020.

We estimated 8,017 excess influenza cases (95% CI 1,841 - 14,718) between 4 February 2020 and 20 March 2020. This excess is presented stratified by age in Table 1.

Table 1. Number of excess influenza cases in Catalonia from 4 February 2020 to 20 March 2020, after the peak of the seasonal influenza epidemic, and the percentage of all influenza cases in that period that they make up, overall and by age group.

For peer review only

Age group	Estimated number of excess influenza cases (95% CI)	Percentage of all influenza cases in this age group made up by the estimated excess cases (95% CI)
Younger than 15	2,078 (160 - 4,078)	13.6% (1.0% - 26.7%)
Between 15 and 64	4,670 (2,387 - 7,124)	20.9% (10.7% - 31.8%)
Older than 64	142 (33 - 260)	8.9% (2.1% - 16.3%)
Total	8,017 (1,841 - 14,718)	20.4% (4.7% - 37.5%)

Excess influenza cases compared with COVID-19 diagnoses

Figure 4 depicts the number of excess influenza cases and COVID-19 diagnoses each day after the peak of the 2019-2020 seasonal influenza epidemic. Excess influenza cases increased rapidly from 24 February 2020, peaking on 7 March 2020. They steeply declined from 15 March 2020, coinciding with an increase in the number of COVID-19 diagnoses.

There were 4,347 excess influenza cases and 1,497 clinical diagnoses of COVID-19 on 14 March 2020, comparing with just 2,575 excess influenza cases (40% less) and a striking 16,547 (539% increase) clinical diagnoses of COVID-19 on 20 March 2020.

Discussion

In mid-February 2020, we observed an unusually high, larger than expected number of influenza cases in the daily published data. In Catalonia, the 2019-2020 seasonal influenza epidemic reached its peak on 4 February 2020. Based on previous years' data, influenza diagnoses were expected to decrease rapidly over the following weeks. However, the number of influenza diagnoses instead remained stable, which was counterintuitive and inconsistent with data from past influenza seasons. This increase in observed influenza diagnoses over

For peer review only

those expected, here named "excess influenza," correlates over time with the observed number of COVID-19 cases. Excess influenza cases could be used in future for the early detection of competing outbreaks.

Using four of the previous nine influenza seasons as a benchmark, we detected 8,017 excess influenza cases between 4 February 2020 and 20 March 2020. This excess was higher in people aged 15-64 years, with over 20% more cases than expected. The excess started to decrease after 15 March 2020. Worryingly, these results suggest that SARS-CoV-2 could have already been circulating in the Catalan population when the first imported case was reported on 25 February 2020. People infected with COVID-19 may have been masked under ILI diagnoses in primary care, allowing continuing community transmission of COVID-19 before public health measures were taken.

To our knowledge, this is the first study attempting to quantify the start of the COVID-19 epidemic in Spain by comparing the number of reported ILI cases with the expected figures based on previous influenza seasons. The excess influenza cases metric could be useful for monitoring future outbreaks of COVID-19 and other competing viral epidemics.

Our study has several limitations. We used ecological data and modelled it using data from previous seasons, therefore assuming a direct causal link between excess influenza cases and the COVID-19 pandemic. Although we lack confirmatory tests or antigenic data for the estimated excess influenza cases, our results agree with a recent study that tested all influenza samples in Los Angeles for SARS-CoV-2, finding 2.2-10.7% of the tested samples positive for the pathogen.[17]

The observed excess influenza cases could have been due to a panic effect, in which the current coronavirus infodemic, a rapid spread of misinformation, has encouraged people to consult healthcare professionals more frequently and for milder symptoms than usual. However, our data showed that the number of influenza diagnoses dropped drastically and COVID-19 diagnoses increased after 15 March 2020. New COVID-19 guidelines were released on 15 March 2020 in Spain that recommended only testing hospital-admitted patients and healthcare staff and encouraging GPs to diagnose COVID-19 clinically without PCR confirmation.[14] At least some of the excess ILI cases were thus likely to have actually been COVID-19 cases.

Our study also has strengths. The data used were good quality, as demonstrated in many previous publications,[18–24] were obtained directly from primary-care records, and have been validated against gold-standard sentinel systems. This existing database covers over 85% of the population of Catalonia, which allowed us to rapidly detect excess influenza cases across the whole population and in different age groups.

In conclusion, the full extent of the SARS-CoV-2 pandemic is still unknown. The confirmed number of cases may be just the tip of the iceberg, due to the lack of testing of patients

For peer review only

presenting mild COVID-19 symptoms. We need comprehensive, well-designed, seroprevalence studies to know how many people have been infected. The surveillance of excess influenza cases using widely available primary-care electronic medical records could help detect new outbreaks of COVID-19 and other ILI-causing pathogens, supporting early testing and public health responses.

For peer review only

What is already know in this topic
First imported COVID-19 case was reported in Catalonia on 25 February.<br>It is uncertain if SARS-CoV-2 was circulating in the community before or after that.
**What this study adds?**
Influenza cases slowed down before COVID-19 was reported in Catalonia, to then surprisingly increase again.<br>Our study suggests that they were undetected COVID-19 cases masked as influenza diagnoses long before the first community acquired cases were reported.

For peer review only

Copyright

The Corresponding Author has the right to grant on behalf of all authors and does grant on behalf of all authors, an exclusive licence on a worldwide basis to the BMJ Publishing Group Ltd to permit this article (if accepted) to be published in BMJ editions and any other BMJPGL products and sublicences such use and exploit all subsidiary rights, as set out in our licence.

Competing interest statement

All authors have completed the ICMJE uniform disclosure form at www.icmje.org/coi_disclosure.pdf and declare: Dr Prieto-Alhambra reports grants and other from AMGEN; grants, non-financial support and other from UCB Biopharma; grants from Les Laboratoires Servier, outside the submitted work; and Janssen, on behalf of IMI-funded EHDEN and EMIF consortiums, and Synapse Management Partners have supported training programmes organised by DPA's department and open for external participants. APU reports grants from Fundacion Alfonso Martin Escudero and the Medical Research Council. No other relationships or activities that could appear to have influenced the submitted work.

Transparency declaration

Lead authors affirm that the manuscript is an honest, accurate, and transparent account of the study being reported; that no important aspects of the study have been omitted; and that any discrepancies from the study as planned have been explained.

Ethical approval

No ethical approval was required. Analyses were only conducted on de-identified and aggregated data available on the public domain.

Funding and study sponsors

The research was partially supported by the National Institute for Health Research (NIHR) Oxford Biomedical Research Centre (BRC). DPA is funded through an NIHR Senior Research Fellowship (Grant number SRF-2018-11-ST2-004). The views expressed in this publication are those of the author(s) and not necessarily those of the NHS, the National Institute for Health Research or the Department of Health. AP-U is supported by Fundacion Alfonso Martin Escudero and the Medical Research Council (grant numbers MR/K501256/1, MR/N013468/1)

Acknowledgements

The authors acknowledge English language editing by Dr Jennifer A de Beyer of the Centre for Statistics in Medicine, University of Oxford.

Contributorship statement

All authors contributed to the design of the study, the interpretation of the results, and reviewed the manuscript. EC and NM had access to the data, performed the statistical analysis, and acted as guarantors. EC, NM, AP-U, and DP-A wrote the first draft of the manuscript. MM-P and DP-A are joint senior authors. The corresponding author attests that all listed authors meet authorship criteria and that no others meeting the criteria have been omitted

PPI statement

This research was done without patient involvement. Patients were not invited to comment on the study design and were not consulted to develop patient relevant outcomes or interpret the

For peer review only

results. Patients were not invited to contribute to the writing or editing of this document for readability or accuracy.

Data sharing statement

Data on influenza case counts for each season and age group are publicly available in Diagnosticat, in the SeGrip section: https://www.ics.gencat.cat/sisap/grip/principal. COVID-19 case counts are available on reasonable request. No additional data is available.

For peer review only

References

1 European Centre for Disease Prevention and Control. Novel coronavirus disease 2019 (COVID-19) pandemic: increased transmission in the EU/EEA and the UK – sixth update – 12 March 2020. Stockholm: ECDC; 2020.

2 Aylward, Bruce (WHO); Liang W (PRC). Report of the WHO-China Joint Mission on Coronavirus Disease 2019 (COVID-19). *WHO* 2020.

3 Young BE, Ong SWX, Kalimuddin S, *et al.* Epidemiologic Features and Clinical Course of Patients Infected with SARS-CoV-2 in Singapore. *JAMA - J Am Med Assoc* Published Online First: 2020. doi:10.1001/jama.2020.3204

4 Zhou F, Yu T, Du R, *et al.* Clinical course and risk factors for mortality of adult inpatients with COVID-19 in Wuhan, China: a retrospective cohort study. *Lancet* Published Online First: 2020. doi:10.1016/s0140-6736(20)30566-3

5 Huang C, Wang Y, Li X, *et al.* Clinical features of patients infected with 2019 novel coronavirus in Wuhan, China. *Lancet* Published Online First: 2020. doi:10.1016/S0140-6736(20)30183-5

6 Chen N, Zhou M, Dong X, *et al.* Epidemiological and clinical characteristics of 99 cases of 2019 novel coronavirus pneumonia in Wuhan, China: a descriptive study. *Lancet* Published Online First: 2020. doi:10.1016/S0140-6736(20)30211-7

7 Bi Q, Wu Y, Mei S, *et al.* Epidemiology and Transmission of COVID-19 in Shenzhen China: Analysis of 391 cases and 1,286 of their close contacts. *medRxiv* Published Online First: 2020. doi:10.1101/2020.03.03.20028423

8 Bernard Stoecklin S, Rolland P, Silue Y, *et al.* First cases of coronavirus disease 2019 (COVID-19) in France: surveillance, investigations and control measures, January 2020. *Eurosurveillance* 2020;**25**:2000094. doi:10.2807/1560-7917.ES.2020.25.6.2000094

9 Boelle P-Y, Sentinelles syndromic and viral surveillance group D. Excess cases of Influenza like illnesses in France synchronous with COVID19 invasion. *medRxiv* 2020;:2020.03.14.20035741. doi:10.1101/2020.03.14.20035741

10 Torner N, Basile L, Martínez A, *et al.* Assessment of two complementary influenza surveillance systems: Sentinel primary care influenza-like illness versus severe hospitalized laboratory-confirmed influenza using the moving epidemic method. *BMC Public Health* 2019;**19**:1089. doi:10.1186/s12889-019-7414-9

11 Closas P, Coma E, Méndez L. Sequential detection of influenza epidemics by the Kolmogorov-Smirnov test. *BMC Med Inform Decis Mak* 2012;**12**:112. doi:10.1186/1472-6947-12-112

12 SISAP. Grip. Seguiment de la grip a Catalunya.

For peer review only

https://www.ics.gencat.cat/sisap/grip/faq?lang=ct (accessed 30 Mar 2020).

13 Bolíbar B, Fina Avilés F, Morros R, *et al.* Base de datos SIDIAP: La historia clínica informatizada de Atención Primaria como fuente de información para la investigación epidemiológica. *Med Clin (Barc)* 2012;**138**:617–21. doi:10.1016/j.medcli.2012.01.020

14 Guia d'actuació enfront de casos d'infecció pel nou coronavirus SARS-CoV-2.

15 Box GEP, Jenkins GM, Reinsel GC. *Time series analysis: Forecasting and control: Fourth edition*. 2013. doi:10.1002/9781118619193

16 R Core Team. R software: Version 3.5.1. *R Found Stat Comput* Published Online First: 2018. doi:10.1007/978-3-540-74686-7

17 Spellberg B, Haddix M, Lee R, *et al.* Community Prevalence of SARS-CoV-2 Among Patients With Influenzalike Illnesses Presenting to a Los Angeles Medical Center in March 2020. *JAMA* Published Online First: 2020. doi:10.1001/jama.2020.4958

18 Recalde M, Manzano-Salgado CB, Díaz Y, *et al.* Validation of cancer diagnoses in electronic health records: Results from the information system for research in primary care (SIDIAP) in northeast spain. *Clin Epidemiol* 2019;**11**:1015–24. doi:10.2147/CLEP.S225568

19 Ponjoan A, Garre-Olmo J, Blanch J, *et al.* Epidemiology of dementia: prevalence and incidence estimates using validated electronic health records from primary care. *Clin Epidemiol* 2019;**11**:217–28. doi:10.2147/CLEP.S186590

20 Ponjoan A, Garre-Olmo J, Blanch J, *et al.* How well can electronic health records from primary care identify Alzheimer's disease cases? *Clin Epidemiol* 2019;**11**:509–18. doi:10.2147/CLEP.S206770

21 Ramos R, Balló E, Marrugat J, *et al.* Validity for use in research on vascular diseases of the SIDIAP (Information System for the Development of Research in Primary Care): the EMMA study. *Rev Esp Cardiol (Engl Ed)* 2012;**65**:29–37. doi:10.1016/j.recesp.2011.07.017

22 Fina-Aviles F, Medina-Peralta M, Mendez-Boo L, *et al.* The descriptive epidemiology of rheumatoid arthritis in Catalonia: a retrospective study using routinely collected data. *Clin Rheumatol* Published Online First: 2016. doi:10.1007/s10067-014-2801-1

23 Muñoz-Ortego J, Vestergaard P, Rubio JB, *et al.* Ankylosing spondylitis is associated with an increased risk of vertebral and nonvertebral clinical fractures: A population-based cohort study. *J Bone Miner Res* 2014;**29**:1770–6. doi:10.1002/jbmr.2217

24 Pagès-Castell A, Carbonell-Abella C, Avilés FF, *et al.* Burden of osteoporotic fractures in primary health care in Catalonia (Spain): A population-based study. *BMC Musculoskelet Disord* 2012;**13**. doi:10.1186/1471-2474-13-79

For peer review only

Figure Legends

Figure 1. Epidemic curves showing the weekly number of new influenza cases during the influenza seasons from autumn-winter 2010-2011 to autumn-winter 2019-2020 in Catalonia, Spain. Curves in solid lines were similar to the 2019-2020 season and included in further modelling. Curves in dashed lines were not similar to the 2019-2020 season and were excluded from further modelling.

Figure 2. Epidemic curves for the 2019-2020 Catalonia influenza season (solid line) and the four seasons in the past decade with a similar peak number of cases (dotted lines: 2011-2012, 2012-2013, 2013-2014, and 2016-2017), centred on the day of the peak number of cases in each curve.

Figure 3. Observed and expected (with 95% CI) weekly new influenza cases each day after the peak of the 2019-2020 Catalonia influenza season, in the full population and in each age group.

Figure 4. Excess influenza cases and clinically diagnosed COVID-19 cases in Catalonia, Spain, as number of cases in the previous 7-day period, from the peak of the 2019-2020 seasonal influenza epidemic (4 February 2020).

For peer review only

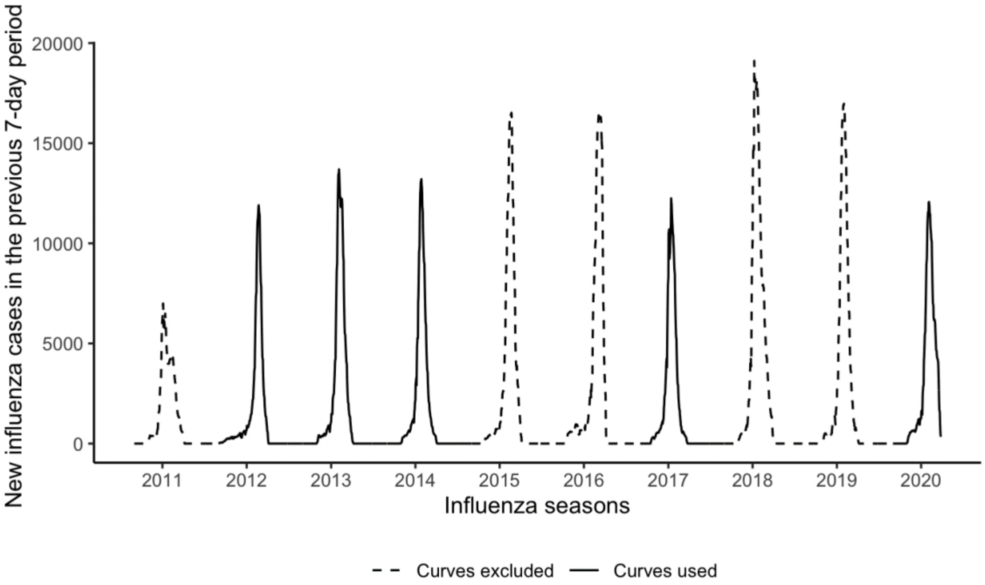

491x304mm (72 x 72 DPI)

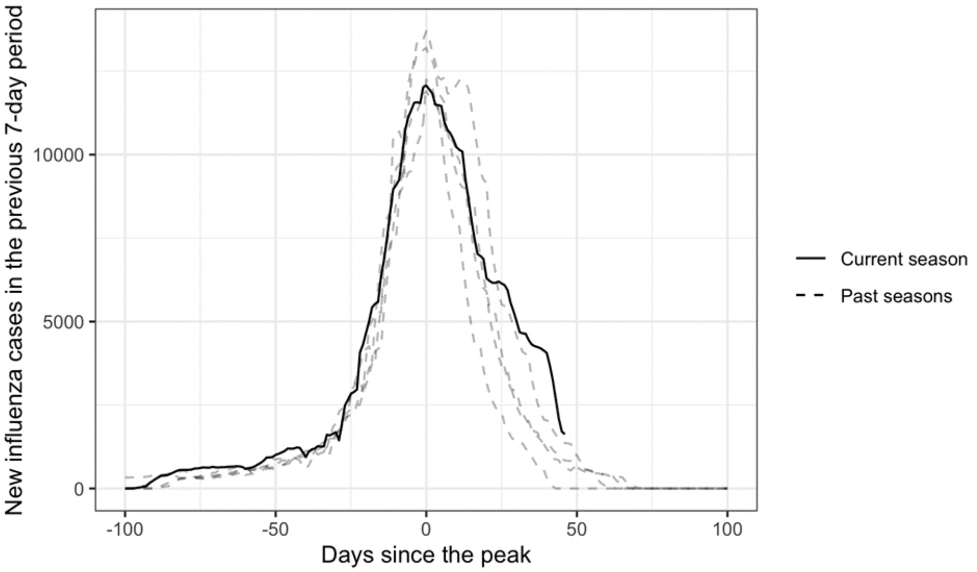

338x209mm (72 x 72 DPI)

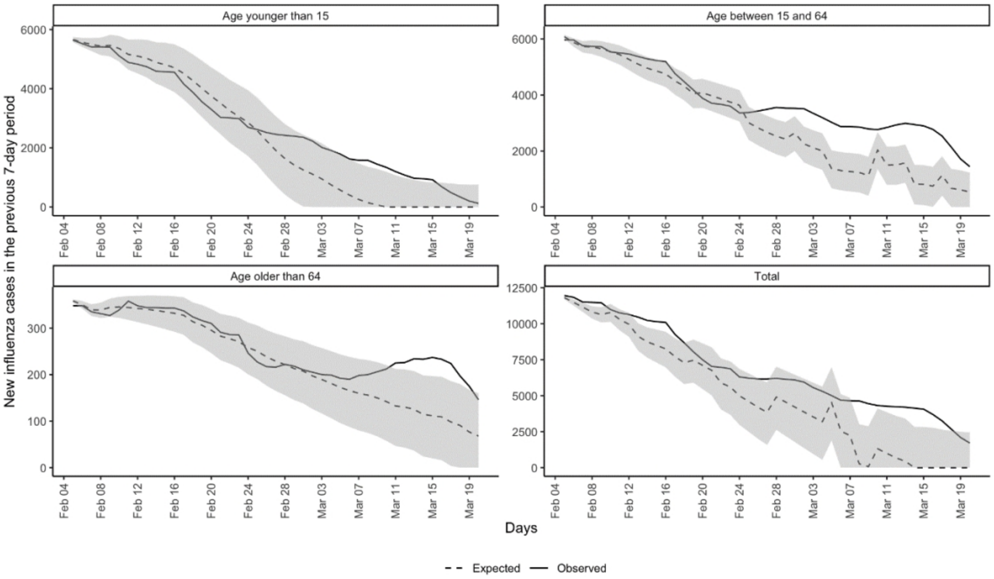

470x291mm (72 x 72 DPI)

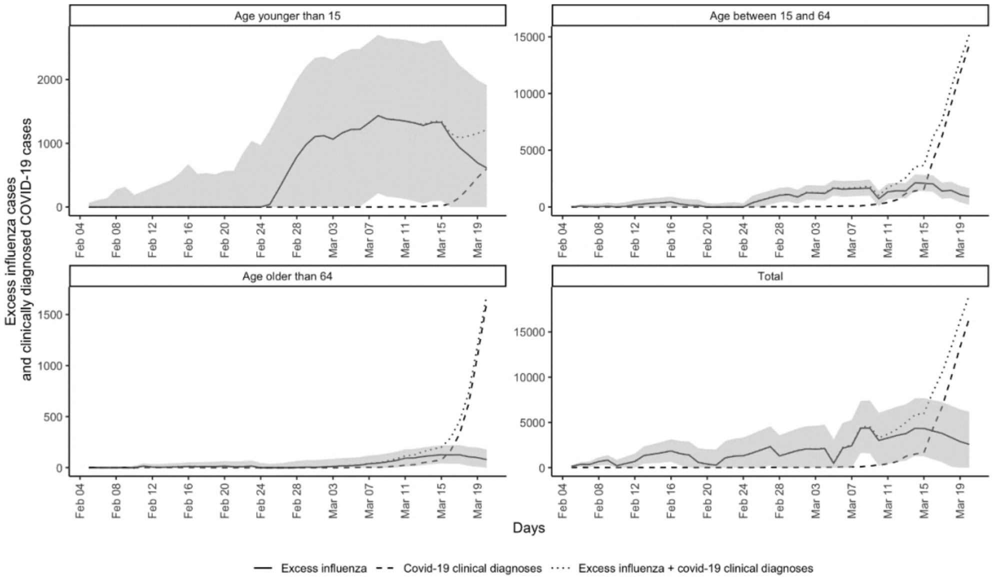

483x299mm (72 x 72 DPI)

Supplementary material

Supplementary Table 1. ICD-10 codes used to identify influenza and COVID-19 cases

Influenza

J10.1	Influenza due to other identified influenza virus with other respiratory manifestations
J10.89	Influenza due to other identified influenza virus with other manifestations
J11.1	Influenza due to unidentified influenza virus with other respiratory manifestations
J11.2	Influenza due to unidentified influenza virus with gastrointestinal manifestations
J11.89	Influenza due to unidentified influenza virus with other manifestations
J10	Influenza due to other identified influenza virus
J11	Influenza due to unidentified influenza virus

COVID-19

B34.2	Coronavirus infection, COVID-19 (with PCR)
B97.29	Coronavirus as the cause of diseases classified elsewhere
B97.21	SARS-associated coronavirus as the cause of diseases classified elsewhere
J12.81	Pneumonia due to SARS-associated coronavirus.

Supplementary Table 2. ARIMA models fitted

Total	ARIMA(1, 1, 2)	$Y_t = 1.86_{t-1}Y_{t-1} + 1.22e_{t-1} - 0.37e_{t-2}$
Age younger than 15	ARIMA(4, 0, 1)	$Y_t = 432.67 + 1.96Y_{t-1} - 1.17Y_{t-2} + 0.35Y_{t-3} - 1.60Y_{t-4} + + 0.59e_{t-1}$
Age between 15 and 64	RIMA(1, 0, 3)	$Y_t = 759.6 + 0.96Y_{t-1} + 0.10e_{t-1} - 0.17e_{t-2} + 0.06e_{t-3}$
Age older than 64	ARIMA(1, 1, 2)	$Y_t = 0.49Y_{t-1} + 0.70 - 0.27e_{t-2}$

For peer review only

STROBE Statement—checklist of items that should be included in reports of observational studies

	Item No	Recommendation	Page No
**Title and abstract**	1	(*a*) Indicate the study's design with a commonly used term in the title or the abstract	1
		(*b*) Provide in the abstract an informative and balanced summary of what was done and what was found	2
**Introduction**			
Background/rationale	2	Explain the scientific background and rationale for the investigation being reported	3
Objectives	3	State specific objectives, including any prespecified hypotheses	3
**Methods**			
Study design	4	Present key elements of study design early in the paper	4
Setting	5	Describe the setting, locations, and relevant dates, including periods of recruitment, exposure, follow-up, and data collection	4
Participants	6	(*a*) *Cohort study*—Give the eligibility criteria, and the sources and methods of selection of participants. Describe methods of follow-up<br>*Case-control study*—Give the eligibility criteria, and the sources and methods of case ascertainment and control selection. Give the rationale for the choice of cases and controls<br>*Cross-sectional study*—Give the eligibility criteria, and the sources and methods of selection of participants	4
		(*b*) *Cohort study*—For matched studies, give matching criteria and number of exposed and unexposed<br>*Case-control study*—For matched studies, give matching criteria and the number of controls per case	-
Variables	7	Clearly define all outcomes, exposures, predictors, potential confounders, and effect modifiers. Give diagnostic criteria, if applicable	4
Data sources/ measurement	8*	For each variable of interest, give sources of data and details of methods of assessment (measurement). Describe comparability of assessment methods if there is more than one group	*4*
Bias	9	Describe any efforts to address potential sources of bias	4
Study size	10	Explain how the study size was arrived at	4
Quantitative variables	11	Explain how quantitative variables were handled in the analyses. If applicable, describe which groupings were chosen and why	4
Statistical methods	12	(*a*) Describe all statistical methods, including those used to control for confounding	4-5
		(*b*) Describe any methods used to examine subgroups and interactions	5
		(*c*) Explain how missing data were addressed	-
		(*d*) *Cohort study*—If applicable, explain how loss to follow-up was addressed<br>*Case-control study*—If applicable, explain how matching of cases and controls was addressed<br>*Cross-sectional study*—If applicable, describe analytical methods taking account of sampling strategy	-
		(*e*) Describe any sensitivity analyses	

Continued on next page

For peer review only - http://bmjopen.bmj.com/site/about/guidelines.xhtml

**Results**			
Participants	13*	(a) Report numbers of individuals at each stage of study—eg numbers potentially eligible, examined for eligibility, confirmed eligible, included in the study, completing follow-up, and analysed	5
		(b) Give reasons for non-participation at each stage	-
		(c) Consider use of a flow diagram	-
Descriptive data	14*	(a) Give characteristics of study participants (eg demographic, clinical, social) and information on exposures and potential confounders	-
		(b) Indicate number of participants with missing data for each variable of interest	-
		(c) *Cohort study*—Summarise follow-up time (eg, average and total amount)	-
Outcome data	15*	*Cohort study*—Report numbers of outcome events or summary measures over time	-
		Case-control study—Report numbers in each exposure category, or summary measures of exposure	-
		Cross-sectional study—Report numbers of outcome events or summary measures	-
Main results	16	(*a*) Give unadjusted estimates and, if applicable, confounder-adjusted estimates and their precision (eg, 95% confidence interval). Make clear which confounders were adjusted for and why they were included	6-7
		(*b*) Report category boundaries when continuous variables were categorized	-
		(*c*) If relevant, consider translating estimates of relative risk into absolute risk for a meaningful time period	6-7
Other analyses	17	Report other analyses done—eg analyses of subgroups and interactions, and sensitivity analyses	6-7
**Discussion**			
Key results	18	Summarise key results with reference to study objectives	7
Limitations	19	Discuss limitations of the study, taking into account sources of potential bias or imprecision. Discuss both direction and magnitude of any potential bias	8
Interpretation	20	Give a cautious overall interpretation of results considering objectives, limitations, multiplicity of analyses, results from similar studies, and other relevant evidence	7-8
Generalisability	21	Discuss the generalisability (external validity) of the study results	8
**Other information**			
Funding	22	Give the source of funding and the role of the funders for the present study and, if applicable, for the original study on which the present article is based	11

*Give information separately for cases and controls in case-control studies and, if applicable, for exposed and unexposed groups in cohort and cross-sectional studies.

Note: An Explanation and Elaboration article discusses each checklist item and gives methodological background and published examples of transparent reporting. The STROBE checklist is best used in conjunction with this article (freely available on the Web sites of PLoS Medicine at http://www.plosmedicine.org/, Annals of Internal Medicine at http://www.annals.org/, and Epidemiology at http://www.epidem.com/). Information on the STROBE Initiative is available at www.strobe-statement.org.

For peer review only

BMJ Open

Excess cases of influenza and the coronavirus epidemic in Catalonia: a time series analysis of primary care electronic medical records covering over 6 million people

Journal:	*BMJ Open*
Manuscript ID	bmjopen-2020-039369.R1
Article Type:	Original research
Date Submitted by the Author:	01-Jul-2020
Complete List of Authors:	Coma Redon, Ermengol; ICS, Sistemes d'Informació dels Serveis d'Atenció Primària (SISAP); IDIAP Jordi Gol<br>Mora, Nuria; ICS, Sistemes d'Informació dels Serveis d'Atenció Primària (SISAP); IDIAP Jordi Gol<br>Prats-Uribe, Albert; University of Oxford, Nuffield Department of Orthopaedics Rheumatology and Musculoskeletal Science<br>Fina Avilés, Francesc; ICS, Sistemes d'Informació dels Serveis d'Atenció Primària (SISAP); IDIAP Jordi Gol<br>Prieto-Alhambra, Daniel; University of Oxford, NDORMS; IDIAP Jordi Gol<br>Medina, Manuel; ICS, Sistemes d'Informació dels Serveis d'Atenció Primària (SISAP); IDIAP Jordi Gol
**Primary Subject Heading**:	Epidemiology
Secondary Subject Heading:	Infectious diseases, General practice / Family practice, Public health
Keywords:	Epidemiology < INFECTIOUS DISEASES, PRIMARY CARE, Public health < INFECTIOUS DISEASES

SCHOLARONE™
Manuscripts

BMJ

I, the Submitting Author has the right to grant and does grant on behalf of all authors of the Work (as defined in the below author licence), an exclusive licence and/or a non-exclusive licence for contributions from authors who are: i) UK Crown employees; ii) where BMJ has agreed a CC-BY licence shall apply, and/or iii) in accordance with the terms applicable for US Federal Government officers or employees acting as part of their official duties; on a worldwide, perpetual, irrevocable, royalty-free basis to BMJ Publishing Group Ltd ("BMJ") its licensees and where the relevant Journal is co-owned by BMJ to the co-owners of the Journal, to publish the Work in this journal and any other BMJ products and to exploit all rights, as set out in our licence.

The Submitting Author accepts and understands that any supply made under these terms is made by BMJ to the Submitting Author unless you are acting as an employee on behalf of your employer or a postgraduate student of an affiliated institution which is paying any applicable article publishing charge ("APC") for Open Access articles. Where the Submitting Author wishes to make the Work available on an Open Access basis (and intends to pay the relevant APC), the terms of reuse of such Open Access shall be governed by a Creative Commons licence – details of these licences and which Creative Commons licence will apply to this Work are set out in our licence referred to above.

Other than as permitted in any relevant BMJ Author's Self Archiving Policies, I confirm this Work has not been accepted for publication elsewhere, is not being considered for publication elsewhere and does not duplicate material already published. I confirm all authors consent to publication of this Work and authorise the granting of this licence.

For peer review only

"Excess cases of influenza and the coronavirus epidemic in Catalonia: a time series analysis of primary care electronic medical records covering over 6 million people"

Ermengol Coma [1,2] *, Nuria Mora [1,2] *, Albert Prats-Uribe [3]*, Francesc Fina [1,2], Daniel Prieto-Alhambra[2,3] §, and Manuel Medina-Peralta [1,2]

1- Sistemes d'Informació dels Serveis d'Atenció Primària (SISAP), Institut Català de la Salut(ICS), Barcelona, Spain.
2- Fundació Institut Universitari per a la recerca a l'Atenció Primària de Salut Jordi Gol i Gurina (IDIAPJGol), Barcelona, Spain.
3- Pharmaco- and Device Epidemiology, Centre for Statistics in Medicine - Nuffield Department of Orthopaedics, Rheumatology, and Musculoskeletal Sciences, University of Oxford, UK

*** joint first authors**

§ Corresponding author:
Prof Daniel Prieto-Alhambra
daniel.prietoalhambra@ndorms.ox.ac.uk
Botnar Research Centre, Windmill Road, OX37LD,
Oxford, United Kingdom

For peer review only

Abstract

Objectives: There is uncertainty about when the first cases of COVID-19 appeared in Spain. We aimed to determine whether influenza diagnoses masked early COVID-19 cases and estimate numbers of undetected COVID-19 cases.

Design: Time-series study of influenza and COVID-19 cases, 2010 to 2020.

Setting: Primary care, Catalonia, Spain.

Participants: People registered in primary-care practices, covering >6 million people and >85% of the population.

Main outcome measures: Weekly new cases of influenza and COVID-19 clinically diagnosed in primary care.

Analyses:

Daily counts of both cases were computed using the total cases recorded over the previous 7 days to avoid weekly effects. Epidemic curves were characterised for the 2010-2011 to 2019-2020 influenza seasons. Influenza seasons with a similar epidemic curve and peak case number as the 2019-2020 season were used to model expected case numbers with ARIMA models, overall and stratified by age. Daily excess influenza cases were defined as the number of observed minus expected cases.

Results:

Four influenza season curves (2011-2012, 2012-2013, 2013-2014, and 2016-2017) were used to estimate the number of expected cases of influenza in 2019-2020. Between 4 February 2020 and 20 March 20202, 8,017 (95% CI: 1,841 to 14,718) excess influenza cases were identified. This excess was highest in the 15-64 age group.

Conclusions:

COVID-19 cases may have been present in the Catalan population when the first imported case was reported on 25 February 2020. COVID-19 carriers may have been misclassified as influenza diagnoses in primary care, boosting community transmission before public health measures were taken. The use of clinical codes could misrepresent the true occurrence of the disease. Serological or PCR testing should be used to confirm these findings. In future, this surveillance of excess influenza could help detect new outbreaks of COVID-19 or other influenza-like pathogens, to initiate early public health responses.

Key words: COVID-19, Influenza , SARS-CoV-2, surveillance

For peer review only

Article summary

Strengths and limitations of this study

- We used good quality data covering >6 million people and >85% of the Catalan population, obtained directly from primary record records.

- Data had previously been validated against gold-standard influenza sentinel systems.

- We used ecological data and modelled it using data from previous seasons, therefore assuming a direct link between excess influenza cases and the COVID-19 pandemic.

- Excess influenza cases could also have been due to a panic effect, where current coronavirus epidemic, had encouraged people to consult healthcare professionals more frequently and for milder symptoms than usual.

- We lack confirmatory tests or antigenic data for the estimated excess influenza cases, but our results agree with the proportion of influenza samples that tested positive for SARS-CoV-2 in a recent study.

For peer review only

Background

A new infectious disease, now named COVID-19, was identified by Chinese authorities on 7 January 2020 as the cause of an outbreak of pneumonia in Wuhan.[1] Caused by SARS-CoV-2, COVID-19 is asymptomatic or presymptomatic in a high proportion of patients, with estimates around 15 to 30%. [2–4] Most patients present mild influenza-like symptoms, including fever, dry cough, fatigue, sore throat, dyspnoea, headache, and myalgia.[5,6] Around 15-20% of symptomatic cases present severe forms of disease that require hospital admission.[1,7] Older people, men, and those with multiple comorbidities appear more likely to suffer more serious types of COVID-19.[5,6,8–10] Conversely, children seem to have a similar probability of infection, but milder and often asymptomatic forms of the disease.[11]

Cases of COVID-19 have grown exponentially and have been reported all over the world. The first three cases in Europe were reported in France on 24 January 2020.[12] The first imported COVID-19 case in Spain was dated 31 January 2020 in La Gomera, and the first in Catalonia reported a month after, on 25 February 2020. The total number of confirmed cases in Catalonia then increased exponentially, with 715 cumulative cases reported by 14 March 2020 and a striking 4,203 on 20 March 2020. Despite these official figures, it is uncertain whether SARS-CoV-2 was circulating in the community before the first official cases. It is difficult to believe, for example, that this airborne infection did not cross the uncontrolled borders between Catalonia and France for a whole month. Some have thus speculated that undetected COVID-19 cases may have been categorised as influenza before the first official case was reported in Spain.[13]

Catalonia is fortunate to have a reliable system for influenza surveillance in place. A network of 60 sentinel general practitioners covering 1% of the total population report daily cases of influenza-like illness (ILI) and take samples for differential diagnosis and confirmation of influenza infections in the region.[14] A specialised hospital-based system takes samples from severe hospitalised flu cases.[14] A community-based surveillance system called Diagnosticat also extracts counts of ILI diagnoses from a network of GP health records in real-time, covering 85% of the population.[15] This last approach allows us to examine trends with granularity and to stratify analyses by age and other factors.

As the first cases of SARS-CoV-2 appeared in Catalonia during the influenza epidemic season and the disease shares some symptomatology with influenza, we hypothesised that SARS-CoV-2 could have been circulating in the community before the first confirmed case, resulting in an excess of influenza diagnoses. We aimed to estimate the number of excess influenza cases in Catalonia, globally and by age, and to examine its relationship with the number of clinically diagnosed COVID-19 cases.

For peer review only

Methods

We used a time-series study of influenza and COVID-19 cases. We extracted data from primary-care electronic medical records covering about 85% of the population of Catalonia, around 6 million people. The study period included all influenza seasons from autumn-winter 2010-2011 to autumn-winter 2019-2020.

The key study outcomes were diagnoses of influenza and COVID-19. Daily frequency of influenza cases recorded in primary-care records were obtained from electronic medical records, as is routinely done for the Diagnosticat database.[16]

Diagnosticat is a website that reports in real-time all influenza diagnoses recorded by all general practitioners working at any of the primary-care centres run by the Institut Català de la Salut (ICS). ICS is the main primary-care health service provider in Catalonia and covers about 85% of practices in the region, who all use the same electronic medical record software, ECAP.[17] Diagnosticat includes all clinical influenza diagnosis codes (ICD-10 codes in Supplementary Table 1) and is updated from ECAP daily (since 2010). It presents the frequency of daily influenza cases and the weekly incidence rates per 10^5 population, a unit that allows diagnoses to be compared between territories independently of the number of inhabitants. Influenza data on Diagnosticat has been shown to accurately represent that in a gold-standard source, the sentinel network of influenza infection reports dataset.[15]

The number of COVID-19 clinical diagnoses were extracted and aggregated using the same data source and methods as for influenza diagnoses. Clinical diagnoses of COVID-19 have been recorded in ECAP since 27 February 2020, when bespoke codes were introduced (Supplementary Table 1). Since March 15 2020, Catalan policies have advocated for cases to be defined based on symptoms alone, with serological or PCR confirmation only required when patients are admitted to hospital or are healthcare staff.[18]

Statistical analysis

Daily counts of influenza and COVID-19 cases were computed based on the frequency of cases recorded in the previous 7-day period to avoid weekly effects on recording practice. All influenza seasons in the study period (2010-2011 to 2019-2020) were analysed separately to characterise annual epidemic curves for seasonal influenza.

Influenza seasons with a visually similar epidemic curve and similar peak case number to that of the 2019-2020 season were selected to model predictions for 2019-2020. We selected these specific seasons after an assessment of the number of cases at the peak to maximise comparability with the current flu season before the COVID-19 outbreak. Auto Regressive Integrated Moving Average (ARIMA) models [19] were fitted to the seasons included in the

For peer review only

analysis for the whole population and for three age groups, paedriatic patients (under 15), adults (15-64), and elderly (over 64 years old).

From the fitted time series, the expected speed of decrease in the number of weekly influenza cases for the 2019-2020 influenza season was calculated for each day after the peak. The expected speed of decrease was defined as the difference between the number of influenza diagnoses predicted between the current day t and the previous day t-1, divided by the number of diagnoses predicted for the previous day t-1 (($cases_t$($cases_{t-1}$)-1). Expected influenza cases were calculated using the sequence $G_t = G_0 * \prod^{t}_{k=1} V_k$, where G_t was the expected influenza cases in the period t, G_0 was the number of cases at the peak and V_k the speed of decrease at day k.

The expected influenza cases for each day on the 2019-2020 season were calculated from the day of the season peak to 20 March 2020, the day of the data extraction. Excess influenza cases were defined as the number of observed minus expected cases, estimated daily as above. We calculated 95% confidence intervals for each estimate. All analyses were performed in R, version 3.5.1. [20]

We further tested our method, as a sensitivity analysis, with data from the most recent (2018-2019) season as a negative control. We checked whether the method was able to identify the season as a “regular” flu season not detecting excess influenza cases.

Patient and public involvement

This research was done without patient involvement. Patients were not invited to comment on the study design and were not consulted to develop patient relevant outcomes or interpret the results. Patients were not invited to contribute to the writing or editing of this document for readability or accuracy.

Results

Previous influenza epidemic curves

Four of the previous nine influenza season curves (2011-2012, 2012-2013, 2013-2014, and 2016-2017) had an epidemic curve and number of influenza cases during the peak similar to the 2019-2020 season, as shown in Figure 1. These four curves were used to estimate the number of expected cases of influenza in 2019-2020. The mean peak number of cases in the included and excluded seasons was 12,762 and 14,680 respectively. The peak number of cases in 2019-2020 was 12,066.

For peer review only

ARIMA models were fitted using the included seasons. Supplementary Table 2 shows the full modelling process and the fitted parameters.

2019-2020 influenza epidemic description

In Catalonia, the 2019-2020 influenza epidemic reached its peak on 4 February 2020, with 12,066 cases in the previous 7 days. Figure 2 shows the evolution of the season compared with past seasons, centred on the day of the peak. By eye, the downwards trend after the peak initially looks very similar to the previous seasons. However, 20 days after the peak, the curve starts to flatten, and the slope slows down. This abnormal pattern in the descending part of the curve differs from the pattern in the previous seasons.

Expected versus observed cases

Figure 3 shows the observed and estimated numbers of weekly new influenza cases (with 95% CI) after the peak of the 2019-2020 influenza season. The estimated expected number of cases were predicted using the selected previous influenza seasons.

In the whole population, observed cases were greater than expected after the seasonal influenza peak, but not always significant during the whole study period. The difference was statistically significant for 23 days between 4 February 2020 and 20 March 2020. Most of these days fell after 8 March 2020, when the difference between observed and expected increased significantly and observed cases remained above the 95% CI band for expected cases for 2 weeks.

There was a greater difference between observed and expected cases among people aged 15-64 years than in both the total population and other age groups, with 25 total days of significant difference. The observed and expected cases diverged earlier than for the total population, separating around 26 February 2020 and remaining significantly different for the rest of the study period.

Observed and expected cases were generally similar in those older than 64 years, until 6 March 2020. Observed cases then quickly rose above expected cases, with the difference becoming significant on 11 March 2020 and remaining so for 9 days, until 19 March 2020.

The shape of the observed cases curve for people younger than 15 years was similar to that for people aged 15-64 years. However, the difference between observed and expected cases was only significantly different for 11 days, between 6 March 2020 and 16 March 2020.

We estimated 8,017 excess influenza cases (95% CI 1,841 - 14,718) between 4 February 2020 and 20 March 2020. This excess is presented stratified by age in Table 1.

For peer review only

Results for our negative control flu season are shown in Supplementary Figure 1. We found no excess influenza in the previous (2018-2019) flu season using the same method.

Table 1. Number of excess influenza cases in Catalonia from 4 February 2020 to 20 March 2020, after the peak of the seasonal influenza epidemic, and the percentage of all influenza cases in that period that they make up, overall and by age group.

Age group	Estimated number of excess influenza cases (95% CI)	Percentage of all influenza cases in this age group made up by the estimated excess cases (95% CI)
Younger than 15	2,078 (160 - 4,078)	13.6% (1.0% - 26.7%)
Between 15 and 64	4,670 (2,387 - 7,124)	20.9% (10.7% - 31.8%)
Older than 64	142 (33 - 260)	8.9% (2.1% - 16.3%)
Total	8,017 (1,841 - 14,718)	20.4% (4.7% - 37.5%)

Excess influenza cases compared with COVID-19 diagnoses

Figure 4 depicts the number of excess influenza cases and COVID-19 diagnoses each day after the peak of the 2019-2020 seasonal influenza epidemic. Excess influenza cases increased rapidly from 24 February 2020, peaking on 7 March 2020. They steeply declined from 15 March 2020, coinciding with an increase in the number of COVID-19 diagnoses. There were 4,347 excess influenza cases and 1,497 clinical diagnoses of COVID-19 on 14 March 2020, comparing with just 2,575 excess influenza cases (40% less) and a striking 16,547 (539% increase) clinical diagnoses of COVID-19 on 20 March 2020.

For peer review only

Discussion

In mid-February 2020, we observed an unusually high, larger than expected number of influenza cases in the daily published data. In Catalonia, the 2019-2020 seasonal influenza epidemic reached its peak on 4 February 2020. Based on previous years' data, influenza diagnoses were expected to decrease rapidly over the following weeks. However, the number of influenza diagnoses instead remained stable, which was counterintuitive and inconsistent with data from past influenza seasons. This increase in observed influenza diagnoses over those expected, here named "excess influenza," correlates over time with the observed number of COVID-19 cases. Excess influenza cases could be used in future for the early detection of competing outbreaks.

Using four of the previous nine influenza seasons as a benchmark, we detected 8,017 excess influenza cases between 4 February 2020 and 20 March 2020. This excess was higher in people aged 15-64 years, with over 20% more cases than expected. The excess started to decrease after 15 March 2020. Worryingly, these results suggest that SARS-CoV-2 could have already been circulating in the Catalan population when the first imported case was reported on 25 February 2020. People infected with COVID-19 may have been masked under ILI diagnoses in primary care, allowing continuing community transmission of COVID-19 before public health measures were taken.

To our knowledge, this is the first study attempting to quantify the start of the COVID-19 epidemic in Spain by comparing the number of reported ILI cases with the expected figures based on previous influenza seasons. The excess influenza cases metric could be useful for monitoring future outbreaks of COVID-19 and other competing viral epidemics.

Our study has several limitations. We used ecological data and modelled it using data from previous seasons, therefore assuming a direct causal link between excess influenza cases and the COVID-19 pandemic. As our method is based on crude count of flu cases, major changes in denominator and population structure could limit the use of the proposed method, but population age and gender has remained relatively stable in the study period.[21] Our main limitation is the possible misclassification of disease status due to limitations related to the use of clinical codes. We lack serological tests or antigenic data for confirmation, and this should be investigated to confirm our findings. our results agree with a study that tested all influenza samples in Los Angeles for SARS-CoV-2, finding 2.2-10.7% of the tested samples positive for the pathogen, and a CDC report that times the start of limited community transmission round mid-January to February.[22,23]

The observed excess influenza cases could have been due to a panic effect, in which the current coronavirus infodemic, a rapid spread of misinformation, has encouraged people to consult healthcare professionals more frequently and for milder symptoms than usual.

For peer review only

However, our data showed that the number of influenza diagnoses dropped drastically and COVID-19 diagnoses increased after 15 March 2020. New COVID-19 guidelines were released on 15 March 2020 in Spain that recommended only testing hospital-admitted patients and healthcare staff and encouraging GPs to diagnose COVID-19 clinically without PCR confirmation.[18] At least some of the excess ILI cases were thus likely to have actually been COVID-19 cases.

Our study also has strengths. The data used were good quality, as demonstrated in many previous publications,[24–30] were obtained directly from primary-care records, and have been validated against gold-standard sentinel systems. This existing database covers over 85% of the population of Catalonia, which allowed us to rapidly detect excess influenza cases across the whole population and in different age groups.

In conclusion, the full extent of the COVID-19 pandemic is still unknown. The confirmed number of cases may be just the tip of the iceberg, due to the lack of testing of patients presenting mild COVID-19 symptoms. We need comprehensive, well-designed, seroprevalence studies to know how many people have been infected. This novel analysis approach could offer a quantitative approach to population surveillance that may be useful for other institutions/regions/countries and could be easily integrated into current information systems. This surveillance of excess influenza cases using widely available primary-care electronic medical records could help detect new outbreaks of COVID-19 and other ILI-causing pathogens, supporting early testing and public health responses.

For peer review only

Copyright

The Corresponding Author has the right to grant on behalf of all authors and does grant on behalf of all authors, an exclusive licence on a worldwide basis to the BMJ Publishing Group Ltd to permit this article (if accepted) to be published in BMJ editions and any other BMJPGL products and sublicences such use and exploit all subsidiary rights, as set out in our licence.

Competing interest statement

All authors have completed the ICMJE uniform disclosure form at www.icmje.org/coi_disclosure.pdf and declare: Dr Prieto-Alhambra reports grants and other from AMGEN; grants, non-financial support and other from UCB Biopharma; grants from Les Laboratoires Servier, outside the submitted work; and Janssen, on behalf of IMI-funded EHDEN and EMIF consortiums, and Synapse Management Partners have supported training programmes organised by DPA's department and open for external participants. APU reports grants from Fundacion Alfonso Martin Escudero and the Medical Research Council. No other relationships or activities that could appear to have influenced the submitted work.

Transparency declaration

Lead authors affirm that the manuscript is an honest, accurate, and transparent account of the study being reported; that no important aspects of the study have been omitted; and that any discrepancies from the study as planned have been explained.

Ethical approval

No ethical approval was required. Analyses were only conducted on de-identified and aggregated data available on the public domain.

Funding and study sponsors

The research was partially supported by the National Institute for Health Research (NIHR) Oxford Biomedical Research Centre (BRC). DPA is funded through an NIHR Senior Research Fellowship (Grant number SRF-2018-11-ST2-004). The views expressed in this publication are those of the author(s) and not necessarily those of the NHS, the National Institute for Health Research or the Department of Health. AP-U is supported by Fundacion Alfonso Martin Escudero and the Medical Research Council (grant numbers MR/K501256/1, MR/N013468/1)

Acknowledgements

The authors acknowledge English language editing by Dr Jennifer A de Beyer of the Centre for Statistics in Medicine, University of Oxford.

Contributorship statement

EC, NM, AP-U, FF-A, MM-P and DP-A contributed to the design of the study, the interpretation of the results, and reviewed the manuscript. EC and NM had access to the data, performed the statistical analysis, and acted as guarantors. EC, NM, AP-U, and DP-A are joint first authors and wrote the first draft of the manuscript. MM-P and DP-A are joint senior authors. The corresponding author attests that all listed authors meet authorship criteria and that no others meeting the criteria have been omitted

PPI statement

This research was done without patient involvement. Patients were not invited to comment on the study design and were not consulted to develop patient relevant outcomes or interpret the

For peer review only

results. Patients were not invited to contribute to the writing or editing of this document for readability or accuracy.

Data sharing statement

Data on influenza case counts for each season and age group are publicly available in Diagnosticat, in the SeGrip section: https://www.ics.gencat.cat/sisap/grip/principal. COVID-19 case counts are available on reasonable request. No additional data is available.

For peer review only

References

1 Ecdc. Novel coronavirus disease 2019 (COVID-19) pandemic: increased transmission in the EU/EEA and the UK – sixth update.

2 Mizumoto K, Kagaya K, Zarebski A, *et al.* Estimating the asymptomatic proportion of coronavirus disease 2019 (COVID-19) cases on board the Diamond Princess cruise ship, Yokohama, Japan, 2020. *Eurosurveillance* 2020;**25**. doi:10.2807/1560-7917.ES.2020.25.10.2000180

3 Kimball A, Hatfield KM, Arons M, *et al.* Asymptomatic and Presymptomatic SARS-CoV-2 Infections in Residents of a Long-Term Care Skilled Nursing Facility — King County, Washington, March 2020. *MMWR Morb Mortal Wkly Rep* 2020;**69**:377–81. doi:10.15585/mmwr.mm6913e1

4 Bianco A, Buckley AB, Overbey J, *et al.* Testing of Patients and Support Persons for Coronavirus Disease 2019 (COVID-19) Infection Before Scheduled Deliveries. *Obstet Gynecol* 2020;Publish Ah. doi:10.1097/AOG.0000000000003985

5 Aylward, Bruce (WHO); Liang W (PRC). Report of the WHO-China Joint Mission on Coronavirus Disease 2019 (COVID-19). *WHO* 2020.

6 Young BE, Ong SWX, Kalimuddin S, *et al.* Epidemiologic Features and Clinical Course of Patients Infected with SARS-CoV-2 in Singapore. *JAMA - J Am Med Assoc* Published Online First: 2020. doi:10.1001/jama.2020.3204

7 PRIETO-ALHAMBRA D, Ballo E, Coma-Redon E, *et al.* Hospitalization and 30-day fatality in 121,263 COVID-19 outpatient cases. *medRxiv* Published Online First: 2020. doi:10.1101/2020.05.04.20090050

8 Zhou F, Yu T, Du R, *et al.* Clinical course and risk factors for mortality of adult inpatients with COVID-19 in Wuhan, China: a retrospective cohort study. *Lancet* Published Online First: 2020. doi:10.1016/s0140-6736(20)30566-3

9 Huang C, Wang Y, Li X, *et al.* Clinical features of patients infected with 2019 novel coronavirus in Wuhan, China. *Lancet* Published Online First: 2020. doi:10.1016/S0140-6736(20)30183-5

10 Chen N, Zhou M, Dong X, *et al.* Epidemiological and clinical characteristics of 99 cases of 2019 novel coronavirus pneumonia in Wuhan, China: a descriptive study. *Lancet* Published Online First: 2020. doi:10.1016/S0140-6736(20)30211-7

11 Bi Q, Wu Y, Mei S, *et al.* Epidemiology and Transmission of COVID-19 in Shenzhen China: Analysis of 391 cases and 1,286 of their close contacts. *medRxiv* Published Online First: 2020. doi:10.1101/2020.03.03.20028423

12 Bernard Stoecklin S, Rolland P, Silue Y, *et al.* First cases of coronavirus disease 2019 (COVID-19) in France: surveillance, investigations and control measures, January

For peer review only

2020. *Eurosurveillance* 2020;**25**:2000094. doi:10.2807/1560-7917.ES.2020.25.6.2000094

13 Boelle P-Y, Sentinelles syndromic and viral surveillance group D. Excess cases of Influenza like illnesses in France synchronous with COVID19 invasion. *medRxiv* 2020;:2020.03.14.20035741. doi:10.1101/2020.03.14.20035741

14 Torner N, Basile L, Martínez A, *et al.* Assessment of two complementary influenza surveillance systems: Sentinel primary care influenza-like illness versus severe hospitalized laboratory-confirmed influenza using the moving epidemic method. *BMC Public Health* 2019;**19**:1089. doi:10.1186/s12889-019-7414-9

15 Closas P, Coma E, Méndez L. Sequential detection of influenza epidemics by the Kolmogorov-Smirnov test. *BMC Med Inform Decis Mak* 2012;**12**:112. doi:10.1186/1472-6947-12-112

16 SISAP. Grip. Seguiment de la grip a Catalunya. https://www.ics.gencat.cat/sisap/grip/faq?lang=ct (accessed 30 Mar 2020).

17 Bolíbar B, Fina Avilés F, Morros R, *et al.* Base de datos SIDIAP: La historia clínica informatizada de Atención Primaria como fuente de información para la investigación epidemiológica. *Med Clin (Barc)* 2012;**138**:617–21. doi:10.1016/j.medcli.2012.01.020

18 Guia d'actuació enfront de casos d'infecció pel nou coronavirus SARS-CoV-2.

19 Box GEP, Jenkins GM, Reinsel GC. *Time series analysis: Forecasting and control: Fourth edition*. 2013. doi:10.1002/9781118619193

20 R Core Team. R software: Version 3.5.1. *R Found Stat Comput* Published Online First: 2018. doi:10.1007/978-3-540-74686-7

21 IDESCAT. Territorial and demographic indicators. Estructura per edats, envelliment i dependència. Catalunya. 2020.https://www.idescat.cat/pub/?id=inddt&n=915&lang=en (accessed 24 Jun 2020).

22 Spellberg B, Haddix M, Lee R, *et al.* Community Prevalence of SARS-CoV-2 Among Patients With Influenzalike Illnesses Presenting to a Los Angeles Medical Center in March 2020. *JAMA* Published Online First: 2020. doi:10.1001/jama.2020.4958

23 Jorden MA, Rudman SL, Villarino E, *et al.* Evidence for Limited Early Spread of COVID-19 Within the United States, January–February 2020. *MMWR Morb Mortal Wkly Rep* 2020;**69**:680–4. doi:10.15585/mmwr.mm6922e1

24 Recalde M, Manzano-Salgado CB, Díaz Y, *et al.* Validation of cancer diagnoses in electronic health records: Results from the information system for research in primary care (SIDIAP) in northeast spain. *Clin Epidemiol* 2019;**11**:1015–24. doi:10.2147/CLEP.S225568

25 Ponjoan A, Garre-Olmo J, Blanch J, *et al.* Epidemiology of dementia: prevalence and incidence estimates using validated electronic health records from primary care. *Clin*

For peer review only

Epidemiol 2019;**11**:217–28. doi:10.2147/CLEP.S186590

26 Ponjoan A, Garre-Olmo J, Blanch J, *et al.* How well can electronic health records from primary care identify Alzheimer's disease cases? *Clin Epidemiol* 2019;**11**:509–18. doi:10.2147/CLEP.S206770

27 Ramos R, Balló E, Marrugat J, *et al.* Validity for use in research on vascular diseases of the SIDIAP (Information System for the Development of Research in Primary Care): the EMMA study. *Rev Esp Cardiol (Engl Ed)* 2012;**65**:29–37. doi:10.1016/j.recesp.2011.07.017

28 Fina-Aviles F, Medina-Peralta M, Mendez-Boo L, *et al.* The descriptive epidemiology of rheumatoid arthritis in Catalonia: a retrospective study using routinely collected data. *Clin Rheumatol* Published Online First: 2016. doi:10.1007/s10067-014-2801-1

29 Muñoz-Ortego J, Vestergaard P, Rubio JB, *et al.* Ankylosing spondylitis is associated with an increased risk of vertebral and nonvertebral clinical fractures: A population-based cohort study. *J Bone Miner Res* 2014;**29**:1770–6. doi:10.1002/jbmr.2217

30 Pagès-Castell A, Carbonell-Abella C, Avilés FF, *et al.* Burden of osteoporotic fractures in primary health care in Catalonia (Spain): A population-based study. *BMC Musculoskelet Disord* 2012;**13**. doi:10.1186/1471-2474-13-79

For peer review only

Figure Legends

Figure 1. Epidemic curves showing the weekly number of new influenza cases during the influenza seasons from autumn-winter 2010-2011 to autumn-winter 2019-2020 in Catalonia, Spain. Curves in solid lines were similar to the 2019-2020 season and included in further modelling. Curves in dashed lines were not similar to the 2019-2020 season and were excluded from further modelling.

Figure 2. Epidemic curves for the 2019-2020 Catalonia influenza season (solid line) and the four seasons in the past decade with a similar peak number of cases (dotted lines: 2011-2012, 2012-2013, 2013-2014, and 2016-2017), centred on the day of the peak number of cases in each curve.

Figure 3. Observed and expected (with 95% CI) weekly new influenza cases each day after the peak of the 2019-2020 Catalonia influenza season, in the full population and in each age group.

Figure 4. Excess influenza cases and clinically diagnosed COVID-19 cases in Catalonia, Spain, as number of cases in the previous 7-day period, from the peak of the 2019-2020 seasonal influenza epidemic (4 February 2020).

For peer review only

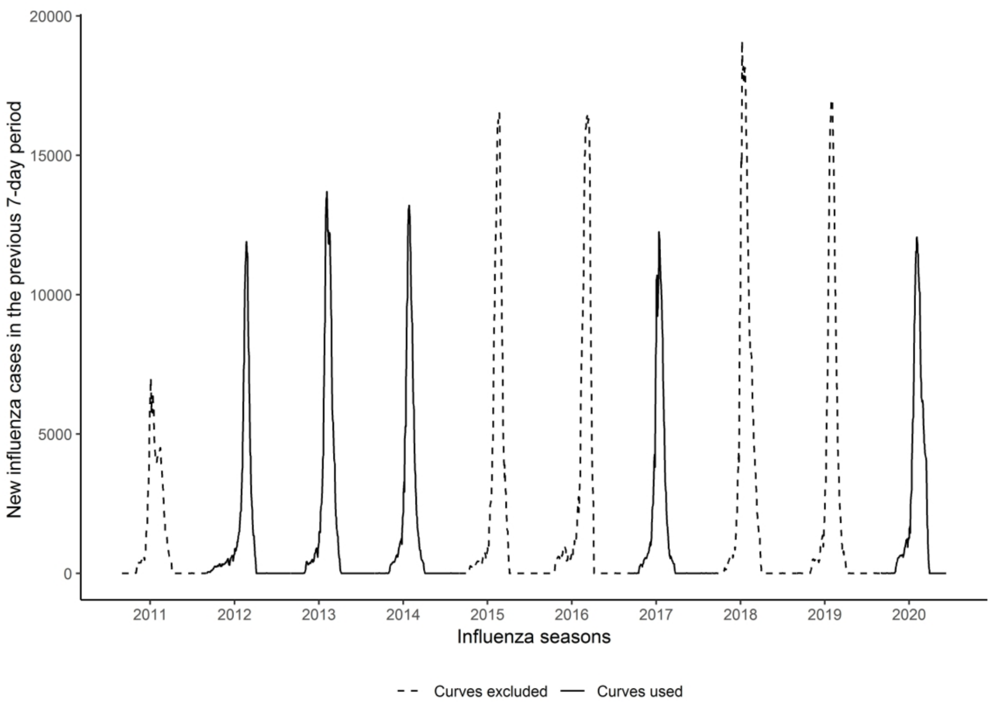

Epidemic curves showing the weekly number of new influenza cases during the influenza seasons from autumn-winter 2010-2011 to autumn-winter 2019-2020 in Catalonia, Spain. Curves in solid lines were similar to the 2019-2020 season and included in further modelling. Curves in dashed lines were not similar to the 2019-2020 season and were excluded from further modelling.

203x152mm (300 x 300 DPI)

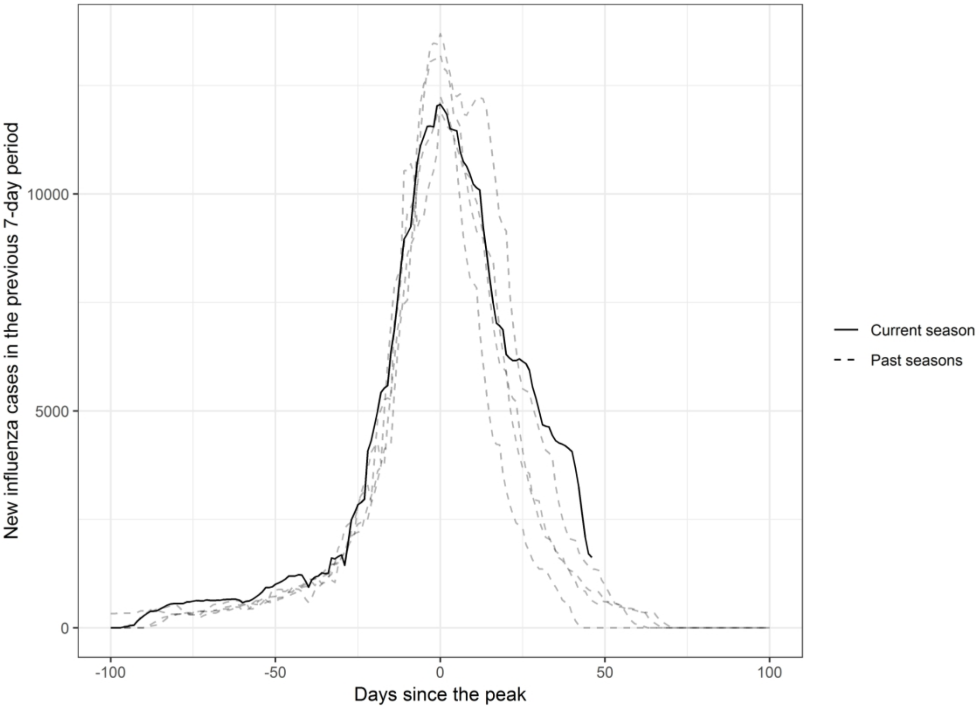

Epidemic curves for the 2019-2020 Catalonia influenza season (solid line) and the four seasons in the past decade with a similar peak number of cases (dotted lines: 2011-2012, 2012-2013, 2013-2014, and 2016-2017), centred on the day of the peak number of cases in each curve.

203x152mm (300 x 300 DPI)

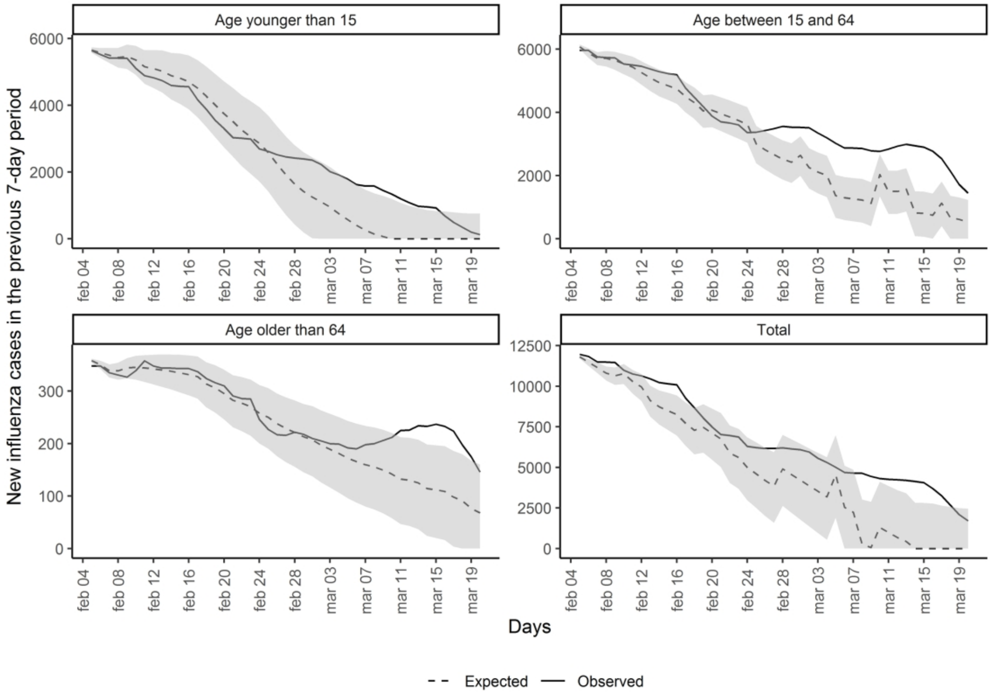

Observed and expected (with 95% CI) weekly new influenza cases each day after the peak of the 2019-2020 Catalonia influenza season, in the full population and in each age group.

203x152mm (300 x 300 DPI)

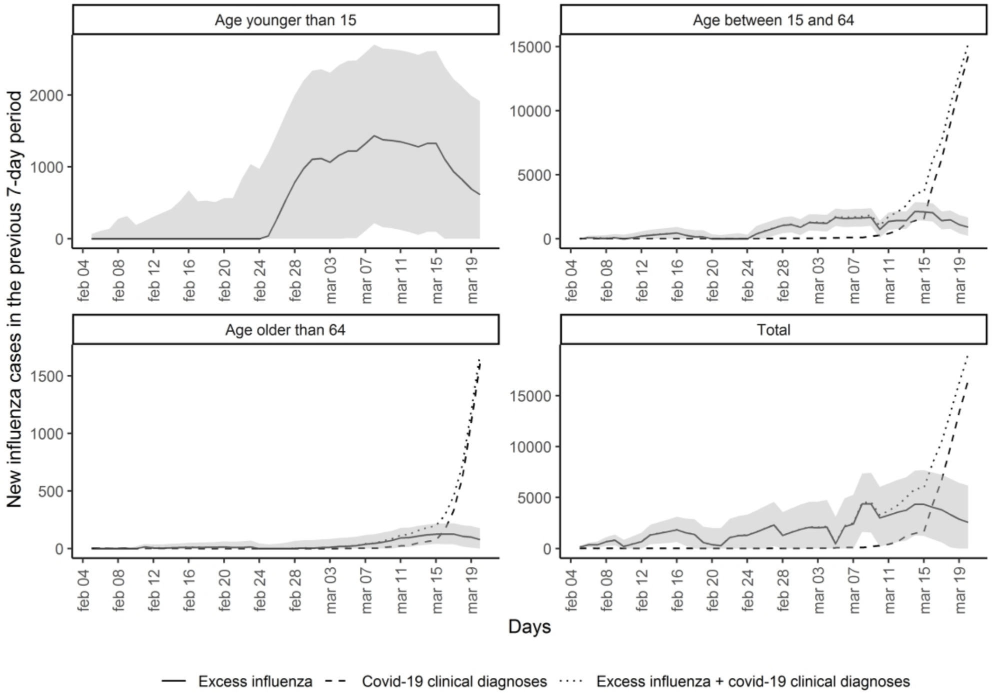

Excess influenza cases and clinically diagnosed COVID-19 cases in Catalonia, Spain, as number of cases in the previous 7-day period, from the peak of the 2019-2020 seasonal influenza epidemic (4 February 2020).

203x152mm (300 x 300 DPI)

Excess cases of influenza suggest an earlier start to the coronavirus epidemic in Spain than official figures tell us: an analysis of primary care electronic medical records from over 6 million people from Catalonia

Supplementary material

For peer review only

Supplementary Table 1. ICD-10 codes used to identify influenza and COVID-19 cases

Influenza

J10.1	Influenza due to other identified influenza virus with other respiratory manifestations
J10.89	Influenza due to other identified influenza virus with other manifestations
J11.1	Influenza due to unidentified influenza virus with other respiratory manifestations
J11.2	Influenza due to unidentified influenza virus with gastrointestinal manifestations
J11.89	Influenza due to unidentified influenza virus with other manifestations
J10	Influenza due to other identified influenza virus
J11	Influenza due to unidentified influenza virus

COVID-19

B34.2	Coronavirus infection, COVID-19 (with PCR)
B97.29	Coronavirus as the cause of diseases classified elsewhere
B97.21	SARS-associated coronavirus as the cause of diseases classified elsewhere
J12.81	Pneumonia due to SARS-associated coronavirus.

Supplementary Table 2. ARIMA models fitted

Total	ARIMA(1, 1, 2)	$Y_t = 1.86_{t-1}Y_{t-1} + 1.22e_{t-1} - 0.37e_{t-2}$
Age younger than 15	ARIMA(4, 0, 1)	$Y_t = 432.67 + 1.96Y_{t-1} - 1.17Y_{t-2} + 0.35Y_{t-3} - 1.60Y_{t-4} + + 0.59e_{t-1}$
Age between 15 and 64	RIMA(1, 0, 3)	$Y_t = 759.6 + 0.96\ Y_{t-1} + 0.10e_{t-1} - 0.17e_{t-2} + 0.06e_{t-3}$
Age older than 64	ARIMA(1, 1, 2)	$Y_t = 0.49\ Y_{t-1} + 0.70 - 0.27e_{t-2}$

For peer review only

Supplementary Figure 1. Observed and expected (with 95% CI) weekly new influenza cases each day after the peak of the 2018-2019 Catalonia influenza season, in the full population and in each age group.

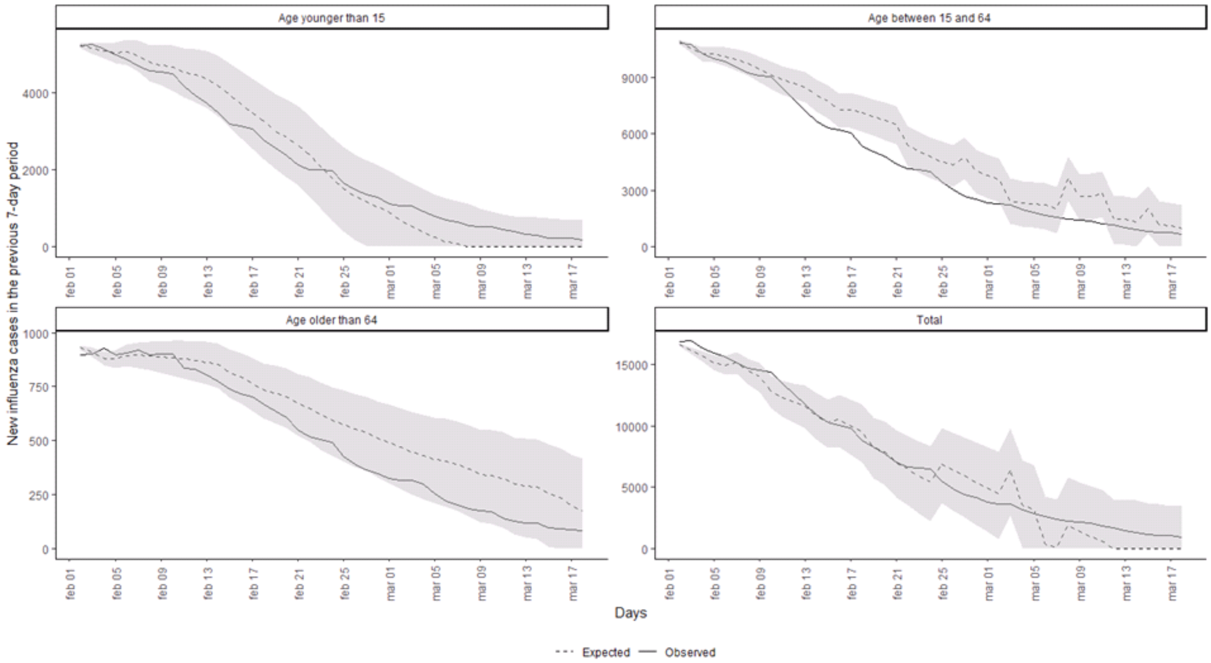

review only

STROBE Statement—checklist of items that should be included in reports of observational studies

	Item No	Recommendation	Page No
**Title and abstract**	1	(*a*) Indicate the study's design with a commonly used term in the title or the abstract	1
		(*b*) Provide in the abstract an informative and balanced summary of what was done and what was found	2
**Introduction**			
Background/rationale	2	Explain the scientific background and rationale for the investigation being reported	3
Objectives	3	State specific objectives, including any prespecified hypotheses	3
**Methods**			
Study design	4	Present key elements of study design early in the paper	4
Setting	5	Describe the setting, locations, and relevant dates, including periods of recruitment, exposure, follow-up, and data collection	4
Participants	6	(*a*) *Cohort study*—Give the eligibility criteria, and the sources and methods of selection of participants. Describe methods of follow-up *Case-control study*—Give the eligibility criteria, and the sources and methods of case ascertainment and control selection. Give the rationale for the choice of cases and controls *Cross-sectional study*—Give the eligibility criteria, and the sources and methods of selection of participants	4
		(*b*) *Cohort study*—For matched studies, give matching criteria and number of exposed and unexposed *Case-control study*—For matched studies, give matching criteria and the number of controls per case	-
Variables	7	Clearly define all outcomes, exposures, predictors, potential confounders, and effect modifiers. Give diagnostic criteria, if applicable	4
Data sources/ measurement	8*	For each variable of interest, give sources of data and details of methods of assessment (measurement). Describe comparability of assessment methods if there is more than one group	*4*
Bias	9	Describe any efforts to address potential sources of bias	4
Study size	10	Explain how the study size was arrived at	4
Quantitative variables	11	Explain how quantitative variables were handled in the analyses. If applicable, describe which groupings were chosen and why	4
Statistical methods	12	(*a*) Describe all statistical methods, including those used to control for confounding	4-5
		(*b*) Describe any methods used to examine subgroups and interactions	5
		(*c*) Explain how missing data were addressed	-
		(*d*) *Cohort study*—If applicable, explain how loss to follow-up was addressed *Case-control study*—If applicable, explain how matching of cases and controls was addressed *Cross-sectional study*—If applicable, describe analytical methods taking account of sampling strategy	-
		(*e*) Describe any sensitivity analyses	

Continued on next page

For peer review only - http://bmjopen.bmj.com/site/about/guidelines.xhtml

**Results**			
Participants	13*	(a) Report numbers of individuals at each stage of study—eg numbers potentially eligible, examined for eligibility, confirmed eligible, included in the study, completing follow-up, and analysed	5
		(b) Give reasons for non-participation at each stage	-
		(c) Consider use of a flow diagram	-
Descriptive data	14*	(a) Give characteristics of study participants (eg demographic, clinical, social) and information on exposures and potential confounders	-
		(b) Indicate number of participants with missing data for each variable of interest	-
		(c) *Cohort study*—Summarise follow-up time (eg, average and total amount)	-
Outcome data	15*	*Cohort study*—Report numbers of outcome events or summary measures over time	-
		Case-control study—Report numbers in each exposure category, or summary measures of exposure	-
		Cross-sectional study—Report numbers of outcome events or summary measures	-
Main results	16	(*a*) Give unadjusted estimates and, if applicable, confounder-adjusted estimates and their precision (eg, 95% confidence interval). Make clear which confounders were adjusted for and why they were included	6-7
		(*b*) Report category boundaries when continuous variables were categorized	-
		(*c*) If relevant, consider translating estimates of relative risk into absolute risk for a meaningful time period	6-7
Other analyses	17	Report other analyses done—eg analyses of subgroups and interactions, and sensitivity analyses	6-7
**Discussion**			
Key results	18	Summarise key results with reference to study objectives	7
Limitations	19	Discuss limitations of the study, taking into account sources of potential bias or imprecision. Discuss both direction and magnitude of any potential bias	8
Interpretation	20	Give a cautious overall interpretation of results considering objectives, limitations, multiplicity of analyses, results from similar studies, and other relevant evidence	7-8
Generalisability	21	Discuss the generalisability (external validity) of the study results	8
**Other information**			
Funding	22	Give the source of funding and the role of the funders for the present study and, if applicable, for the original study on which the present article is based	11

*Give information separately for cases and controls in case-control studies and, if applicable, for exposed and unexposed groups in cohort and cross-sectional studies.

Note: An Explanation and Elaboration article discusses each checklist item and gives methodological background and published examples of transparent reporting. The STROBE checklist is best used in conjunction with this article (freely available on the Web sites of PLoS Medicine at http://www.plosmedicine.org/, Annals of Internal Medicine at http://www.annals.org/, and Epidemiology at http://www.epidem.com/). Information on the STROBE Initiative is available at www.strobe-statement.org.

For peer review only
